# A Novel Small Molecule p53 Stabilizer for Brain Cell Differentiation

**DOI:** 10.3389/fchem.2019.00015

**Published:** 2019-01-31

**Authors:** Joana D. Amaral, Dário Silva, Cecília M. P. Rodrigues, Susana Solá, Maria M. M. Santos

**Affiliations:** Research Institute for Medicines (iMed.ULisboa), Faculty of Pharmacy, Universidade de Lisboa, Lisbon, Portugal

**Keywords:** antiproliferative agents, brain tumor, cell differentiation, p53, spirooxindole

## Abstract

Brain tumor, as any type of cancer, is assumed to be sustained by a small subpopulation of stem-like cells with distinctive properties that allow them to survive conventional therapies and drive tumor recurrence. Thus, the identification of new molecules capable of controlling stemness properties may be key in developing effective therapeutic strategies for cancer by inducing stem-like cells differentiation. Spiropyrazoline oxindoles have previously been shown to induce apoptosis and cell cycle arrest, as well as upregulate p53 steady-state levels, while decreasing its main inhibitor MDM2 in the HCT116 human colorectal carcinoma cell line. In this study, we made modifications in this scaffold by including combinations of different substituents in the pyrazoline ring in order to obtain novel small molecules that could modulate p53 activity and act as differentiation inducer agents. The antiproliferative activity of the synthesized compounds was assessed using the isogenic pair of HCT116 cell lines differing in the presence or absence of the p53 gene. Among the tested spirooxindoles, spiropyrazoline oxindole **1a** was selective against the cancer cell line expressing wild-type p53 and presented low cytotoxicity. This small molecule induced neural stem cell (NSC) differentiation through reduced SOX2 (marker of multipotency) and increased βIII-tubulin (marker of neural differentiation) which suggests a great potential as a non-toxic inducer of cell differentiation. More importantly, in glioma cancer cells (GL-261), compound **1a** reduced stemness, by decreasing SOX2 protein levels, while also promoting chemotherapy sensitization. These results highlight the potential of p53 modulators for brain cell differentiation, with spirooxindole **1a** representing a promising lead molecule for the development of new brain antitumor drugs.

## Introduction

Glioblastoma and malignant gliomas are the most common and lethal primary brain tumors in adults. Following diagnosis, the survival is no longer than 1 year even after surgical resection, radiation, and temozolomide chemotherapy (Tanaka et al., 2013). A major problem in developing more effective treatments for cancer, in general, results from the presence of subpopulations of cancer stem cells (CSCs) with phenotypic similarities to normal stem cells. CSCs have been implied in tumor initiation, invasiveness, and recurrence due to their long-lasting properties and chemotherapy resistance. In fact, while traditional chemotherapy or radiotherapy normally involve the shrinkage of tumors by standard anti-proliferative mechanisms, CSCs are not entirely eliminated by classical approaches, persisting the tumorigenic potential and rapidly giving rise to relapses. Importantly, brain tumors also present subpopulations of CSCs (Vescovi et al., 2006). Therefore, cancer cells may instead be coaxed into becoming normal cells by differentiation therapy eliminating the complete CSC population and sensitizing tumor cells for classical chemotherapy. This, in turn, could be achieved by reactivating endogenous differentiation programs in cancer cells to resume the maturation process and eliminate tumor phenotypes. In this regard, recent reports have highlighted the importance of p53 tumor suppressor in controlling stem cell fate and glioma cell proliferation and invasiveness (Qin et al., 2007; Molchadsky et al., 2010; Zhao and Xu, 2010; Li et al., 2013; Merlino et al., 2018). The first indication that p53 could inhibit neural stem cells (NSCs) self-renewal was observed in p53 knockout mice, in which increased levels of cell self-renewal were detected in the neurogenic niches, when compared with wild-type animals (Meletis et al., 2006). Regarding neural differentiation, we and others have also demonstrated that p53 is an integral component of neurogenesis pathways, promoting neurite outgrowth and axonal regeneration in neural stem cells (NSCs) (Xavier et al., 2014) and primary neurons (Giovanni et al., 2006), and interacting with key regulators of neurogenesis to redirect stem cells to differentiation, as an alternative to cell death (Sola et al., 2012). p53 also inhibits the expression of essential transcription factors responsible for stemness maintenance (Lin et al., 2005; Abdelalim and Tooyama, 2014; Lin and Lin, 2017). Therefore, p53 activation serves as a barrier for induced pluripotent stem cells (iPSCs) tumorigenicity (Aloni-Grinstein et al., 2014). One of the main negative regulators of p53 is the murine double minute 2 protein (MDM2), so the development of small molecule p53–MDM2 interaction inhibitors is a promising approach in drug discovery to reactivate p53 (Zhao et al., 2015; Ribeiro et al., 2016a). Most of these inhibitors contain a rigid heterocyclic scaffold with three lipophilic groups to mimic Phe19, Trp23, and Leu26 of p53. As part of our ongoing efforts in developing spirooxindoles to activate p53 (Ribeiro et al., 2012, 2014, 2016b, 2017; Monteiro et al., 2014), we have recently developed a library of spiropyrazoline oxindoles that induce cell cycle arrest in HCT116 colon cancer cells by partially inhibiting the p53-MDM2 protein-protein interaction in live cells (Nunes et al., 2017). However, the effect of this scaffold on cell differentiation and, consequently, on the regulation of malignant tumor phenotype, was never explored. Since poor differentiation is an important hallmark of cancer cells, the manipulation of p53 for redirecting neural differentiation could emerge as a low toxicity alternative, in comparison with the conventionally used therapies, to sensitize brain tumor cells to chemotherapy (Her et al., 2018). Herein, we report the synthesis of a new library of spiropyrazoline oxindoles with potential to induce p53-mediated neural differentiation and thus to develop efficient differentiation strategies for brain tumors.

## Materials and Methods

### Chemistry

#### General

All reagents and solvents were obtained from commercial suppliers and were used without further purification, except for reaction solvents, which were dried prior to their use. Hydrazonoyl chlorides **3a**–**c** (Wolkoff, 1975; Zhang et al., 2010), and 3-methylene indolinones **2a**–**j** (Sun et al., 1998) were synthesized according to the methods described in the literature. Melting points were determined using a Kofler camera Bock monoscope M and are uncorrected. Merck Silica Gel 60 F_254_ plates were used for analytical TLC. Flash column chromatography was performed on Merck Silica Gel (230–400 mesh). Preparative TLC was performed on Merck Silica Gel 60 GF_254_ over glass plates. ^1^H and ^13^C NMR spectra were recorded on a Bruker 300 Avance at 300 MHz (^1^H NMR) and 75 MHz (^13^C NMR). ^1^H and ^13^C chemical shifts (δ) are expressed in parts per million (ppm) using the solvent as internal reference, and proton coupling constants (J) in hertz (Hz). ^1^H spectral data are reported as follows: chemical shift, multiplicity (d, doublet; dd, doublet of doublets; m, multiplet; s, singlet), coupling constant, and integration. All compounds tested showed purity ≥ 95% by LC-MS, performed on a Waters Alliance 2,695 HPLC with a Waters SunFire C18 column (100 × 2.1 mm; 5 μm) at 20°C, using as mobile phase a solution of acetonitrile with Milli-Q water containing 0.5% formic acid (v/v) (3:7), and employing a photodiode array detector to scan wavelength absorption from 210 to 780 nm. Experiments of mass spectrometry were performed on a Micromass® Quattro Micro triple quadrupole (Waters®, Ireland) with an electrospray in positive ion mode (ESI+), ion source at 120°C, capillary voltage of 3.0 kV and source voltage of 30 V, at the Liquid Chromatography and Mass Spectrometry Laboratory, Faculty of Pharmacy, University of Lisbon.

#### General Procedure for the Synthesis of Spiropyrazoline Oxindoles 1a–r

Triethylamine (3.0 equiv) was added dropwise to a mixture of 3-methylene indolin-2-one **2** (1.0 equiv), and hydrazonoyl chloride **3** (1.2–2.0 equiv) in dry CH_2_Cl_2_ under nitrogen atmosphere. The reaction was stirred at room temperature for 16–24 h. The mixture was then washed with water and the aqueous phase extracted with ethyl acetate (3x). The combined organic extracts were dried over anhydrous Na_2_SO_4_ and the solvent was removed under reduce pressure. The residue obtained was purified by flash chromatography on silica gel using as eluent EtOAc/*n*-Hexane 1:4, and recrystallized from CH_2_Cl_2_/heptane to afford the desired compound.

##### 5′-(tert-butyl)-6-chloro-5-fluoro-2′,4′-diphenyl-2′,4′-dihydrospiro[indoline-3,3′-pyrazol]-2 one (1a)

Following the general procedure, to a solution of **2a** (40 mg, 0.18 mmol) in CH_2_Cl_2_ (10 ml) was added **3a** (1.2 eq) and triethylamine (3 eq). Reaction time: 16 h. White solid (41 mg, 92%). Mp: 240–242°C; ^1^H NMR (300 MHz, CDCl_3_) δ (ppm): 8.14 (s, 1H, NH), 7.46–7.29 (m, 3H, ArH), 7.11–7.05 (m, 2H, ArH), 6.88–6.64 (m, 6H, ArH), 6.00 (d, *J* = 9Hz, 1H, ArH), 4.45 (s, 1H, H-4′), 1.18 (s, 9H, C(CH_3_)_3_); ^13^C NMR (75 MHz, CDCl_3_) δ (ppm): 177.5 (C=O), 161.9 (C=N), 155.7 (d, *J*_C–*F*_ = 243 Hz), 145.5 (Cq), 138.1 (Cq), 136.6 (Cq), 134.6 (Cq), 129.0 (CH), 128.7 (CH), 121.9 (d, *J* = 19.5 Hz), 116.3 (Cq), 115.4 (d, *J* = 24,75 Hz), 111.9 (CH), 77.3 (Cspiro), 62.5 (CH-4′), 34.9 (C(CH_3_)_3_), 29.4 (C(CH_3_)_3_) (Supplementary Datasheet 1); MS (ESI+) m/z calcd for C_26_H_23_ClFN_3_O: 447, found 448 [M + H]^+^.

##### 5′-(tert-butyl)-6-chloro-2′-(4-chlorophenyl)-5-fluoro-4′-phenyl-2′,4′-dihydrospiro[indoline-3,3′-pyrazol]-2-one (1b)

Following the general procedure, to a solution of **2a** (30 mg, 0.09 mmol) in CH_2_Cl_2_ (10 ml) was added **3b** (1.2 eq) and triethylamine (3 eq). Reaction time: 18 h. White solid (21 mg, 70%). Mp: 220–222°C; ^1^H NMR (300 MHz, CDCl_3_) δ (ppm): 8.19 (s, 1H, NH), 7.41–7.29 (m, 4H, ArH), 7.05 (d, *J* = 9 Hz, 2H, ArH), 6.81 (d, *J* = 6 Hz, 1H, ArH), 6.75–6.68 (m, 3H, ArH), 6.01 (d, *J* = 9 Hz, 1H, ArH), 4.46 (s, 1H, H-4′), 1.18 (s, 9H, C(CH_3_)_3_); ^13^C NMR (75 MHz, CDCl_3_) δ (ppm): 177.2 (C=O), 162.6 (C=N), 155.8 (d, *J*_C–*F*_ = 243 Hz), 144.2 (Cq), 136.6 (Cq), 134.2 (Cq), 129.0 (CH), 128.8 (CH), 126.7 (Cq), 125.7 (d, *J* = 7.5 Hz), 122.3 (d, *J* = 19.5 Hz), 117.8 (CH), 115.4 (d, *J* = 25.5 Hz), 112.0 (CH), 77.3 (Cspiro), 62.6 (CH-4′), 34.9 (C(CH_3_)_3_), 29.4 (C(CH_3_)_3_); MS (ESI+) m/z calcd for C_26_H_22_Cl_2_FN_3_O: 481, found 482 [M + H]^+^.

##### 4′-(2-bromophenyl)-6-chloro-2′-(4-chlorophenyl)-5-fluoro-5′-phenyl-2′,4′-dihydrospiro[indoline-3,3′-pyrazol]-2-one (1c)

Following the general procedure, to a solution of **2b** (50 mg, 0.15 mmol) in CH_2_Cl_2_ (10 ml) was added **3c** (1.4 eq) and triethylamine (3 eq). Reaction time: 18 h. White solid (40 mg, 67%). Mp: 241–242°C; ^1^H NMR (300 MHz, CDCl_3_) δ (ppm): 8.80 (s, 1H, NH), 7.63–7.60 (m, 2H, ArH), 7.48 (d, *J* = 6 Hz, 1H, ArH), 7.34–7.29 (m, 3H, ArH), 7.25–7.07 (m, 5H, ArH), 6.93–6.89 (m, 1H, ArH), 6.85 (d, *J* = 9 Hz, 2H, ArH), 6.00 (d, *J* = 9 Hz, 1H, ArH), 5.67 (s, 1H, H-4′); ^13^C NMR (75 MHz, CDCl_3_) δ (ppm): 176.5 (C=O), 162.3 (C=N), 155.2 (d, *J*_C–*F*_ = 263 Hz), 150.0 (Cq), 142.8 (Cq), 137.7 (Cq), 133.52 (Cq), 133.48 (Cq), 130.7 (CH), 129.2 (CH), 128.8 (CH), 126.9 (CH), 125.6 (d, *J* = 15.75 Hz), 117.7 (CH), 115.1 (d, *J* = 26.25 Hz), 112.4 (CH), 77.3 (Cspiro), 60.8 (CH-4′); MS (ESI+) m/z calcd for C_28_H_17_BrCl_2_FN_3_O: 579 found 580 [M + H]^+^.

##### 4′-(2-bromophenyl)-5′-(tert-butyl)-6-chloro-5-fluoro-2′-phenyl-2′,4′-dihydrospiro[indoline-3,3′-pyrazol]-2-one (1d)

Following the general procedure, to a solution of **2b** (40 mg, 0.12 mmol) in CH_2_Cl_2_ (10 ml) was added **3a** (1.4 eq) and triethylamine (3 eq). Reaction time: 18 h. White solid (22 mg, 53%). Mp: 243–245°C; ^1^H NMR (300 MHz, CDCl_3_) δ (ppm): 8.04 (s, 1H, NH), 7.48–7.28 (m, 3H, ArH), 7.21–7.15 (m, 1H, ArH), 7.08 (t, *J* = 9 Hz, 2H, ArH), 6.93–6.80 (m, 4H, ArH), 5.90 (d, *J* = 9 Hz, 1H, ArH), 5.11 (s, 1H, H-4′), 1.20 (s, 9H, C(CH_3_)_3_); ^13^C NMR (75 MHz, CDCl_3_) δ (ppm): 176.8 (C=O), 161.7 (C=N), 155.6 (d, *J*_C–*F*_ = 243.0 Hz), 145.4 (Cq), 137.5 (Cq), 133.7 (Cq), 133.4 (Cq), 130.9 (CH), 130.1 (CH), 129.1 (CH), 127.7 (CH), 125.8 (d, *J* = 7,5 Hz), 121.8 (CH), 116.5 (CH), 114.8 (d, J = 25.5 Hz), 112.0 (CH), 77.3 (Cspiro), 60.2 (CH-4′), 34.8 (C(CH_3_)_3_), 29.4 (C(CH_3_)_3_); MS (ESI+) m/z calcd for C_26_H_22_BrClFN_3_O: 525 found 526 [M + H]^+^.

##### 4′-(2-bromophenyl)-5′-(tert-butyl)-6-chloro-2′-(4-chlorophenyl)-5-fluoro-2′,4′ dihydrospiro[indoline-3,3′- pyrazol]-2-one (1e)

Following the general procedure, to a solution of **2b** (40 mg, 0.12 mmol) in CH_2_Cl_2_ (10 ml) was added **3b** (1.2 eq) and triethylamine (3 eq). Reaction time: 24 h. White solid (15 mg, 30%). Mp: 251–252°C; ^1^H NMR (300 MHz, CDCl_3_) δ (ppm): 7.51 (br s, 1H, NH), 7.49–7.39 (m, 2H, ArH), 7.33–7.30 (m, 1H, ArH), 7.22–7.17 (m, 1H, ArH), 7.05 (d, *J* = 9 Hz, 2H, ArH), 6.85 (d, *J* = 6 Hz, 1H, ArH), 6.75 (d, *J* = 9 Hz, 2H, ArH), 5.88 (d, *J* = 9 Hz, 1H, ArH), 5.12 (s, 1H, H-4′), 1.19 (s, 9H, C(CH_3_)_3_); ^13^C NMR (75 MHz, CDCl_3_) δ (ppm): 175.8 (C=O), 162.4 (C=N), 155.7 (d, *J*_C–*F*_ = 241.5 Hz), 144.1 (Cq), 137.4 (Cq), 133.5 (Cq), 130.8 (CH), 130.3 (CH), 129.0 (CH), 127.7 (CH), 127.1 (CH), 125.8 (CH), 118.2 (CH), 114.9 (d, *J* = 24.75 Hz), 111.8 (CH), 77.3 (Cspiro), 60.3 (CH-4′), 34.8 (C(CH_3_)_3_), 29.4 (C(CH_3_)_3_); MS (ESI+) m/z calcd for C_26_H_21_BrCl_2_FN_3_O: 559 found 560 [M + H]^+^.

##### 4′-(3-bromophenyl)-5′-(tert-butyl)-6-chloro-5-fluoro-2′-phenyl-2′,4′-dihydrospiro[indoline-3,3′-pyrazol]-2-one (1f)

Following the general procedure, to a solution of **2c** (60 mg, 0.16 mmol) in CH_2_Cl_2_ (10 ml) was added **3a** (1.4 eq) and triethylamine (3 eq). Reaction time: 18 h. White solid (30 mg, 52%). Mp: 220–222°C; ^1^H NMR (300 MHz, CDCl_3_) δ (ppm): 7.61 (s, 1H, NH), 7.48–7.44 (m, 2H, ArH), 7.13–7.06 (m, 3H, ArH), 6.89–6.75 (m, 5H, ArH), 6.10 (d, *J* = 9 Hz, 1H, ArH), 4.40 (s, 1H, H-4′), 1.18 (s, 9H, C(CH_3_)_3_); ^13^C NMR (75 MHz, CDCl_3_) δ (ppm): 177.4 (C=O), 161.3 (C=N), 155.8 (d, *J*_C–*F*_ = 243.75 Hz), 145.2 (Cq), 136.9 (Cq), 131.8 (CH), 129.1 (CH), 125.5 (Cq), 123.2 (CH), 121.9 (CH), 116.4 (CH), 115.2 (d, *J* = 23.25 Hz), 113.6 (Cq), 112.3 (CH), 77.3 (Cspiro), 61.9 (CH-4′), 34.9 (C(CH_3_)_3_), 29.4 (C(CH_3_)_3_); MS (ESI+) m/z calcd for C_26_H_22_BrClFN_3_O: 525 found 526 [M + H]^+^.

##### 4′-(3-bromophenyl)-5′-(tert-butyl)-6-chloro-2′-(4-chlorophenyl)-5-fluoro-2′,4′-dihydrospiro[indoline-3,3′-pyrazol]-2-one (1g)

Following the general procedure, to a solution of **2c** (60 mg, 0.16 mmol) in CH_2_Cl_2_ (10 ml) was added **3b** (1.4 eq) and triethylamine (3 eq). Reaction time: 17 h. White solid (68 mg, 72%). Mp: 201–203°C; ^1^H NMR (300 MHz, CDCl_3_) δ (ppm): 8.37 (s, 1H, NH), 7.48–7.45 (m, 2H, ArH), 7.11–7.01 (m, 4H, ArH), 6.84 (d, *J* = 6 Hz, 1H, ArH), 6.75–6.62 (m, 3H, ArH), 6.05 (d, *J* = 9 Hz, 1H, ArH), 4.39 (s, 1H, H-4′), 1.18 (s, 9H, C(CH_3_)_3_); ^13^C NMR (75 MHz, CDCl_3_) δ (ppm): 177.1 (C=O), 162.0 (C=N), 155.8 (d, *J*_C−F_ = 243.75 Hz), 143.9 (Cq), 142.2 (Cq), 136.6 (Cq), 132.0 (CH), 129.1 (CH), 125.1 (d, *J* = 8.25 Hz), 123.2 (CH), 117.8 (CH), 115.2 (d, *J* = 32.25 Hz), 113.6 (Cq), 112.4 (CH), 77.2 (Cspiro), 61.9 (CH-4′), 35.0 (C(CH_3_)_3_), 29.4 (C(CH_3_)_3_); MS (ESI+) m/z calcd for C_26_H_21_BrCl_2_FN_3_O: 559 found 560 [M + H]^+^.

##### 4′-(4-bromophenyl)-5′-(tert-butyl)-6-chloro-5-fluoro-2′-phenyl-2′,4′-dihydrospiro[indoline-3,3′-pyrazol]-2-one (1h)

Following the general procedure, to a solution of **2d** (20 mg, 0.06 mmol) in CH_2_Cl_2_ (10 ml) was added **3a** (1.2 eq) and triethylamine (3 eq). Reaction time: 17 h. White solid (14 mg, 65%). Mp: 209–211°C; ^1^H NMR (300 MHz, CDCl_3_) δ (ppm): 9.69 (s, 1H, NH), 7.56–7.35 (m, 4H, ArH), 7.10–7.04 (m, 3H, ArH), 6.85–6.77 (m, 3H, ArH), 6.15 (d, *J* = 9 Hz, 1H, ArH), 4.81 (s, 1H, H-4′), 1.17 (s, 9H, C(CH_3_)_3_); ^13^C NMR (75 MHz, CDCl_3_) δ (ppm): 176.7 (C=O), 162.0 (C=N), 155.9 (d, *J*_C–*F*_ = 240 Hz), 146.6 (Cq), 139.0 (Cq), 132.2 (CH), 129.6 (CH), 127.2 (d, *J* = 7.5 Hz), 123.3 (CH), 121.9 (CH), 116.8 (CH), 115.7 (d, *J* = 24.75 Hz), 112.7 (CH), 77.5 (Cspiro), 62.2 (CH-4′), 35.5 (C(CH_3_)_3_), 29.8 (C(CH_3_)_3_); MS (ESI+) m/z calcd for C_26_H_22_BrClFN_3_O: 525 found 526 [M + H]^+^.

##### 6-chloro-2′-(4-chlorophenyl)-5-fluoro-4′-(2-fluorophenyl)-5′-phenyl-2′,4′-dihydrospiro[indoline-3,3′-pyrazol]-2-one (1i)

Following the general procedure, to a solution of **2e** (30 mg, 0.09 mmol) in CH_2_Cl_2_ (10 ml) was added **3c** (1.2 eq) and triethylamine (3 eq). After 17 h of reaction was added more 0.8 eq of **3c**. Reaction time: 22 h. White solid (25 mg, 83%). Mp: 243–245°C; ^1^H NMR (300 MHz, CDCl_3_) δ (ppm): 7.88 (s, 1H, NH), 7.67–7.63 (m, 2H, ArH), 7.33–7.31 (m, 4H, ArH), 7.11–7.07 (m, 4H, ArH), 6.92–6.82 (m, 4H, ArH), 6.07 (d, *J* = 9 Hz, 1H, ArH), 5.51 (s, 1H, H-4′); ^13^C NMR (75 MHz, CDCl_3_) δ (ppm): 176.4 (C=O), 161.3 (C=N), 155.8 (d, *J*_*C–F*_ = 256.5 Hz), 130.6 (Cq), 129.3 (CH), 129.0 (CH), 128.6 (Cq), 126.7 (CH), 125.1 (d, *J* = 6.75 Hz), 124.6 (Cq), 122.6 (d, *J*_C–*F*_ = 21.75 Hz), 121.4 (CH), 117.0 (CH), 114.9 (d, *J* = 24.75 Hz), 112.1 (CH), 77.2 (Cspiro), 54.6 (CH-4′); MS (ESI+) m/z calcd for C_28_H_17_Cl_2_F_2_N_3_O: 519 found 520 [M + H]^+^.

##### 5′-(tert-butyl)-6-chloro-5-fluoro-4′-(2-fluorophenyl)-2′-phenyl-2′,4′-dihydrospiro[indoline-3,3′-pyrazol]-2-one (1j)

Following the general procedure, to a solution of **2e** (60 mg, 0.19 mmol) in CH_2_Cl_2_ (10 ml) was added **3a** (1.2 eq) and triethylamine (3 eq). Reaction time: 18 h. White solid (66 mg, 74%). Mp: 210–211°C; ^1^H NMR (300 MHz, CDCl_3_) δ (ppm): 8.65 (s, 1H, NH), 7.24–7.12 (m, 3H, ArH), 7.02–6.97 (m, 2H, ArH), 6.88–6.82 (m, 1H, ArH), 6.77–6.70 (m, 4H, ArH), 5.84 (d, *J* = 9 Hz, 1H, ArH), 4.81 (s, 1H, H-4′), 1.20 (s, 9H, C(CH_3_)_3_); ^13^C NMR (75 MHz, CDCl_3_) δ (ppm): 177.5 (C=O), 161.7 (d, *J*_C–*F*_ = 246 Hz), 160.4 (C=N), 155.6 (d, *J*_C–*F*_ = 242.25 Hz), 145.3 (Cq), 137.0 (Cq), 130.6 (d, *J* = 8.25 Hz), 130.4 (d, *J* = 3 Hz), 129.1 (CH), 125.6 (d, *J* = 6.75 Hz), 124.3 (CH), 122.1 (Cq), 121.7 (CH), 116.2 (d, *J* = 21 Hz), 116.0 (CH), 114.6 (d, *J* = 26.25 Hz), 112.4 (CH), 77.3 (Cspiro), 53.3 (CH-4′), 34.8 (C(CH_3_)_3_), 29.3 (C(CH_3_)_3_); MS (ESI+) m/z calcd for C_26_H_22_ClF_2_N_3_O: 465 found 466 [M + H]^+^.

##### 5′-(tert-butyl)-6-chloro-5-fluoro-4′-(4-fluorophenyl)-2′-phenyl-2′,4′-dihydrospiro[indoline-3,3′-pyrazol]-2-one (1k)

Following the general procedure, to a solution of **2f** (30 mg, 0.10 mmol) in CH_2_Cl_2_ (10 ml) was added **3a** (1.2 eq) and triethylamine (3 eq). After 16 h of reaction was added more 0.8 eq of **3a**. Reaction time: 21 h. White solid (20 mg, 66%). Mp: 216–218°C; ^1^H NMR (300 MHz, CDCl_3_) δ (ppm): 8.65 (s, 1H, NH), 8.15 (s, 1H, NH), 7.12–7.02 (m, 4H, ArH), 6.87–6.75 (m, 5H, ArH), 6.05 (d, *J* = 9 Hz, 1H, ArH), 4.43 (s, 1H, H-4′), 1.18 (s, 9H, C(CH_3_)_3_); ^13^C NMR (75 MHz, CDCl_3_) δ (ppm): 177.4 (C=O), 161.8 (C=N), 161.1 (d, *J*_C–*F*_ = 255 Hz), 155.7 (d, *J*_C–*F*_ = 243 Hz), 145.3 (Cq), 143.8 (Cq), 136.6 (Cq), 130.4 (d, *J* = 3 Hz), 129.0 (CH), 125.9 (d, *J* = 7.5 Hz), 124.3 (CH), 122.2 (Cq), 121.8 (CH), 120.1 (Cq), 116.4 (CH), 115.3 (d, *J* = 24.75 Hz), 113.6 (CH), 112.1 (CH), 77.3 (Cspiro), 61.6 (CH-4′), 34.9 (C(CH_3_)_3_), 29.4 (C(CH_3_)_3_); MS (ESI+) m/z calcd for C_26_H_22_ClF_2_N_3_O: 465 found 466 [M + H]^+^.

##### 6-chloro-2′-(4-chlorophenyl)-5-fluoro-4′-(4-fluorophenyl)-5′-phenyl-2′,4′-dihydrospiro[indoline-3,3′-pyrazol]-2-one (1l)

Following the general procedure, to a solution of **2f** (40 mg, 0.16 mmol) in CH_2_Cl_2_ (10 ml) was added **3c** (1.2 eq) and triethylamine (3 eq). After 17 h of reaction was added more 0.8 eq of **3c**. Reaction time: 23 h. White solid (36 mg, 67%). Mp: 217–219°C; ^1^H NMR (300 MHz, CDCl_3_) δ (ppm): 8.01 (s, 1H, NH), 7.63–7.60 (m, 2H, ArH), 7.32–7.28 (m, 3H, ArH), 7.10 (d, *J* = 9 Hz, 2H, ArH), 6.95–6.82 (m, 7H, ArH), 6.21 (d, *J* = 9 Hz, 1H, ArH), 5.14 (s, 1H, H-4′); ^13^C NMR (75 MHz, CDCl_3_) δ (ppm): 177.0 (C=O), 161.5 (C=N), 155.2 (d, *J*_C–*F*_ = 264.75 Hz), 149.7 (Cq), 146.9 (Cq), 136.3 (Cq), 130.9 (d, *J* = 7.5 Hz), 129.5 (CH), 129.2 (CH), 128.7 (CH), 127.0 (Cq), 125.9 (d, *J* = 13.5 Hz), 123.3 (CH), 122.5 (Cq), 121.8 (CH), 120.1 (Cq), 117.2 (CH), 116.4 (d, *J* = 21 Hz), 115.5 (d, *J* = 25.5 Hz), 112.3 (CH), 77.3 (Cspiro), 62.2 (CH-4′); MS (ESI+) m/z calcd for C_28_H_17_Cl_2_F_2_N_3_O: 519 found 520 [M + H]^+^.

##### 5′-(tert-butyl)-6-chloro-4′-(3-chlorophenyl)-2′-(4-chlorophenyl)-5-fluoro-2′,4′-dihydrospiro[indoline-3,3′-pyrazol]-2-one (1m)

Following the general procedure, to a solution of **2g** (40 mg, 0.13 mmol) in CH_2_Cl_2_ (10 ml) was added **3a** (1.2 eq) and triethylamine (3 eq). Reaction time: 17 h. White solid (31 mg, 73%). Mp: 231–232°C; ^1^H NMR (300 MHz, CDCl_3_) δ (ppm): 8.17 (s, 1H, NH), 7.37–7.29 (m, 2H, ArH), 7.12–7.00 (m, 3H, ArH), 6.88–6.69 (m, 5H, ArH), 6.08 (d, *J* = 9 Hz, 1H, ArH), 4.41 (s, 1H, H-4′), 1.19 (s, 9H, C(CH_3_)_3_); ^13^C NMR (75 MHz, CDCl_3_) δ (ppm): 177.3 (C=O), 161.4 (C=N), 155.8 (d, *J*_C–*F*_ = 243.75 Hz), 145.2 (Cq), 136.6 (Cq), 135.1 (Cq), 129.1 (CH), 125.2 (d, *J* = 7.5 Hz), 121.9 (CH), 118.0 (CH), 116.5 (CH), 115.3 (d, *J* = 22.5 Hz), 112.2 (CH), 77.3 (Cspiro), 61.9 (CH-4′), 34.9 (C(CH_3_)_3_), 29.4 (C(CH_3_)_3_); MS (ESI+) m/z calcd for C_26_H_22_Cl_2_FN_3_O: 481 found 482 [M + H]^+^.

##### 5′-(tert-butyl)-6-chloro-2′,4′-bis(4-chlorophenyl)-5-fluoro-2′,4′-dihydrospiro[indoline-3,3′-pyrazol]-2-oneone (1n)

Following the general procedure, to a solution of **2h** (20 mg, 0.08 mmol) in CH_2_Cl_2_ (10 ml) was added **3b** (1.2 eq) and triethylamine (3 eq). Reaction time: 19 h. White solid (35 mg, 85%). Mp: 128–130°C; ^1^H NMR (300 MHz, CDCl_3_) δ (ppm): 8.43 (s, 1H, NH), 7.43–7.31 (m, 2H, ArH), 7.23–7.12 (m, 1H, ArH), 7.04 (d, *J* = 9 Hz, 2H, ArH), 6.83 (d, *J* = 6 Hz, 1H, ArH), 6.77–6.69 (m, 3H, ArH), 6.04 (d, *J* = 9 Hz, 1H, ArH), 4.41 (s, 1H, H-4′), 1.16 (s, 9H, C(CH_3_)_3_); ^13^C NMR (75 MHz, CDCl_3_) δ (ppm): 177.7 (C=O), 162.4 (C=N), 155.8 (d, *J*_C–*F*_ = 243.75 Hz), 144.0 (Cq), 136.8 (Cq), 134.8 (Cq), 132.2 (Cq), 129.3 (d, *J* = 3.75 Hz), 129.0 (CH), 127.0 (CH), 125.3 (d, *J* = 7.5 Hz), 122.6 (d, *J* = 19.5 Hz), 118.0 (CH), 115.3 (d, *J* = 27 Hz), 112.2 (CH), 77.2 (Cspiro), 61.7 (CH-4′), 35.0 (C(CH_3_)_3_), 29.4 (C(CH_3_)_3_); MS (ESI+) m/z calcd for C_26_H_21_Cl_3_FN_3_O: 515 found 516 [M + H]^+^.

##### 5′-(tert-butyl)-6-chloro-4′-(4-chlorophenyl)-5-fluoro-2′-phenyl-2′,4′-dihydrospiro[indoline-3,3′-pyrazol]-2-one (1o)

Following the general procedure, to a solution of **2h** (30 mg, 0.09 mmol) in CH_2_Cl_2_ (10 ml) was added **3a** (1.2 eq) and triethylamine (3 eq). Reaction time: 19 h. White solid (12 mg, 42%). Mp: 220–221°C; ^1^H NMR (300 MHz, CDCl_3_) δ (ppm): 8.80 (s, 1H, NH), 7.41–7.33 (m, 1H, ArH), 7.20–7.05 (m, 3H, ArH), 6.88–6.66 (m, 5H, ArH), 6.08 (d, *J* = 9 Hz, 1H, ArH), 4.42 (s, 1H, H-4′), 1.17 (s, 9H, C(CH_3_)_3_); ^13^C NMR (75 MHz, CDCl_3_) δ (ppm): 177.1 (C=O), 161.6 (C=N), 155.8 (d, *J*_C–*F*_ = 243.75 Hz), 145.3 (Cq), 136.7 (Cq), 134.7 (Cq), 133.1 (Cq), 129.1 (CH), 125.7 (d, *J* = 7.5 Hz), 122.3 (Cq), 121.9 (CH), 116.6 (CH), 115.3 (d, *J* = 24.75 Hz), 112.1 (CH), 77.3 (Cspiro), 61.7 (CH-4′), 34.9 (C(CH_3_)_3_), 29.4 (C(CH_3_)_3_); MS (ESI+) m/z calcd for C_26_H_22_Cl_2_FN_3_O: 481 found 482 [M + H]^+^.

##### 6-chloro-4′-(3-chloro-2-fluorophenyl)-2′-(4-chlorophenyl)-5-fluoro-5′-phenyl-2′,4′-dihydrospiro[indoline-3,3′-pyrazol]-2-one (1p)

Following the general procedure, to a solution of **2i** (50 mg, 0.15 mmol) in CH_2_Cl_2_ (10 ml) was added **3c** (1.2 eq) and triethylamine (3 eq). After 17 h of reaction was added more 0.8 eq of **3c**. Reaction time: 22 h. White solid (62 mg, 75%). Mp: 236–237°C; ^1^H NMR (300 MHz, CDCl_3_) δ (ppm): 8.70 (s, 1H, NH), 7.67–7.63 (m, 2H, ArH), 7.35–7.28 (m, 4H, ArH), 7.11 (d, *J* = 9 Hz, 2H, ArH), 7.02–6.92 (m, 3H, ArH), 6.83 (d, *J* = 9 Hz, 2H, ArH), 6.08 (d, 1H, *J* = 9 Hz, ArH); 5.51 (s, 1H, H-4′); ^13^C NMR (75 MHz, CDCl_3_) δ (ppm): 177.1 (C=O), 163.5 (C=N), 157.7 (d, *J*_C–*F*_ = 247.5 Hz), 155.8 (d, *J*_C–*F*_ = 268.5 Hz), 148.1 (Cq), 142.4 (Cq), 137.0 (Cq), 131.2 (CH), 130.6 (Cq), 129.7 (CH), 129.3 (CH), 128.9 (Cq), 127.2 (Cq), 126.8 (CH), 125.1 (d, *J* = 4.5 Hz), 124.7 (d, *J* = 6 Hz), 123.4 (d, *J* = 38.35 Hz), 123.2 (Cq), 122.0 (d, *J* = 17.25 Hz), 117.0 (CH), 114.7 (d, *J* = 24.75 Hz), 112.9 (CH), 76.2 (Cspiro), 54.8 (CH-4′); MS (ESI+) m/z calcd for C_28_H_16_Cl_3_F_2_N_3_O: 553 found 554 [M + H]^+^.

##### 5′-(tert-butyl)-6-chloro-4′-(3-chloro-2-fluorophenyl)-2′-phenyl-2′,4′-dihydrospiro[indoline-3,3′-pyrazol]-2-one (1q)

Following the general procedure, to a solution of **2j** (30 mg, 0.09 mmol) in CH_2_Cl_2_ (10 ml) was added **3a** (1.1 eq) and triethylamine (3 eq). Reaction time: 16 h. White solid (49 mg, 92%). Mp: 231–232°C; ^1^H NMR (300 MHz, CDCl_3_) δ (ppm): 7.85 (s, 1H, NH), 7.37–7.31 (m, 1H, ArH), 7.24–7.04 (m, 4H, ArH), 6.86–6.78 (m, 4H, ArH), 7.11 (dd, *J* = 6 Hz, 3 Hz, 1H, ArH), 6.07 (d, *J* = 9 Hz, 1H, ArH), 4.86 (s, 1H, H-4′), 1.21 (s, 9H, C(CH_3_)_3_); ^13^C NMR (75 MHz, CDCl_3_) δ (ppm): 176.7 (C=O), 160.5 (C=N), 157.3 (d, *J*_C–*F*_ = 247.5 Hz), 145.3 (Cq), 144.7 (Cq), 135.8 (Cq), 130.7 (CH), 129.0 (CH), 128.9 (CH), 126.6 (CH), 124.4 (d, *J*_C–*F*_ = 4.5 Hz), 124.3 (d, *J*_C–*F*_ = 14.25 Hz), 123.5 (Cq), 122.4 (CH), 121.9 (CH), 116.6 (CH), 111.2 (CH), 77.3 (Cspiro), 53.3 (CH-4′), 34.8 (C(CH_3_)_3_), 29.3 (C(CH_3_)_3_); MS (ESI+) m/z calcd for C_26_H_22_Cl_2_FN_3_O: 481 found 482 [M + H]^+^.

##### 5′-(tert-butyl)-6-chloro-4′-(3-chloro-2-fluorophenyl)-2′-(4-chlorophenyl)-2′,4′-dihydrospiro[indoline-3,3′-pyrazol]-2-one (1r)

Following the general procedure, to a solution of **2j** (30 mg, 0.09 mmol) in CH_2_Cl_2_ (10 ml) was added **3b** (1.1 eq) and triethylamine (3 eq). Reaction time: 16 h. White solid (43 mg, 76%). Mp: 209–211°C; ^1^H NMR (300 MHz, (CD_3_)_2_CO) δ (ppm): 9.83 (s, 1H, NH), 7.53–7.48 (m, 1H, ArH), 7.37–7.24 (m, 2H, ArH), 7.09 (d, *J* = 12 Hz, 2H, ArH), 7.02 (d, *J* = 6 Hz, 1H, ArH), 6.80 (d, *J* = 12 Hz, 2H, ArH), 6.68 (dd, *J* = 6 Hz, *J* = 3 Hz, 1H, ArH), 6.14 (d, *J* = 9 Hz, 1H, ArH), 4.96 (s, 1H, H-4′), 1.20 (s, 9H, C(CH_3_)_3_); ^13^C NMR (75 MHz, (CD_3_)_2_CO) δ (ppm): 176.2 (C=O), 161.2 (C=N), 158.0 (d, *J*_C–*F*_ = 246 Hz), 145.4 (Cq), 144.4 (Cq), 136.3 (Cq), 131.7 (CH), 130.2 (CH), 129.5 (CH), 127.5 (CH), 126.3 (Cq), 126.1 (d, *J*_C–*F*_ = 5.25 Hz), 125.3 (d, *J*_C–*F*_ = 15 Hz), 122.4 (Cq), 122.0 (d, *J* = 18.75 Hz), 118.1 (CH), 111.9 (CH), 76.9 (Cspiro), 54.5 (CH-4′), 35.4 (C(CH_3_)_3_), 29.5 (C(CH_3_)_3_); MS (ESI+) m/z calcd for C_26_H_21_Cl_3_FN_3_O: 515 found 516 [M + H]^+^.

### Biology: General Conditions

#### Cell Culture

HCT116 p53 wild-type (p53^+/+^) and null (p53^−/−^) isogenic cell lines were obtained from GRCF Cell Center and Biorepository (Johns Hopkins University, School of Medicine, Baltimore, MD, USA). The GL-261 mouse glioblastoma cell line was kindly provided by Dr. Dora Brites (University of Lisbon). HCT116 cell lines were grown in McCoy's medium and GL-261 cells in Dulbecco's modified Eagle's medium (DMEM) (both from Gibco, Thermo Fisher Scientific, Waltham, MA, USA). Media were supplemented with 10% fetal bovine serum (FBS) (Gibco) and 1% penicillin/streptomycin solution (Sigma-Aldrich, St Louis, MO, USA). Neural stem cells (NSCs) were derived from 14.5-dpc mouse fetal forebrain. This cell line was established using a method that produces pure cultures of adherent NSCs, which continuously expand by symmetrical division and are capable of tripotential differentiation (Pratt et al., 2000; Silva et al., 2006). NSCs were grown in monolayer and routinely maintained in undifferentiation medium, Euromed-N medium (EuroClone S.p.A., Pavia, Italy), supplemented with 1% N-2 supplement (Gibco), 20 ng/mL epidermal growth factor (EGF; PeproTech EC, London, UK), 20 ng/mL basic fibroblast growth factor (bFGF; PeproTech EC), and 1% penicillin-streptomycin (Gibco, Thermo Fisher Scientific). In differentiating conditions, NSCs were grown in Euromed-N medium supplemented with 1% B-27, 10 ng/mL bFGF, 1% penicillin/streptomycin solution (Sigma-Aldrich) and 0.5% N-2. Cell lines were maintained in a humidified atmosphere of 5% CO_2_, at 37°C. HCT-116 and GL-261 cells were seeded in 96-well plates at 10 × 10^3^ cells/well for cell viability assays. Additionally, NSCs were seeded at 1.8 × 10^4^ cells/well in 96-well plates for cell viability, at 3.0 × 10^5^ cells/well in 6-well plates for flow cytometry, and at 8.2 × 10^5^ cells/dish in 60-mm dishes for Western blot analysis.

#### Cell Treatment

Compound stock solutions were prepared in sterile dimethyl sulfoxide (DMSO, Sigma-Aldrich). After seeding, cells were allowed to adhere for 24 h before exposure to test compounds, which were diluted in culture medium at the indicated concentration and time point. All experiments were performed in parallel with DMSO vehicle control. The final DMSO concentration did not exceed 0.8% (v/v).

#### Cell Viability

Cell viability was assessed 72 h after compound incubation in HCT116 cells and 24 and 48 h after compound incubation in NSCs and GL-261 cells using the CellTiter96® AQueous Non-Radioactive Cell Proliferation Assay (Promega Corporation, Madison, WI, USA), according to the manufacturer's instructions. This colorimetric assay is based on the bio-reduction of 3-(4,5-dimethylthiazo-2- yl)-5-(3-carboxymethoxyphenyl)-2-(4-sulfophenyl)-2H-tetrazolium inner salt (MTS) to water-soluble formazan by dehydrogenase enzymes found within metabolically active cells. The amount of formazan can then be measured through readings at 490 nm absorbance, correlating with the number of living cells in culture. Changes in absorbance were assessed using a GloMax® Multi Detection System (Sunnyvale, CA, USA).

#### Total Protein Extraction and Quantification

NCSs and GL-261 cells were exposed to compound **1a** at 12.5 and 25 μM, or vehicle control (DMSO), for 24 and 48 h. After that, adherent cells were collected, centrifuged, and the pellet resuspended in lysis buffer (1% NP-40, 20 mM Tris-HCl pH 7.4, 150 mM NaCl, 5 mM EDTA, 10% Glycerol, 1 mM dithiothreitol (DTT), and 1X proteases and phosphatases inhibitors) for total protein extraction. Finally, cell lysates were sonicated and centrifuged at 3,200 *g* for 10 min at 4°C. Total protein extracts contained in the supernatants were recovered and stored at −80°C.

Protein concentration was determined by the colorimetric Bradford method using the Bio-Rad Protein Assay reagent (Bio-Rad), according to the manufacturer's instructions. Bovine serum albumine (BSA) (Sigma-Aldrich) was used as standard, and absorbance measurements were performed at 595 nm using GloMax-Multi+ Detection System (Promega).

#### Western Blot and Immunocytochemistry Analysis

Steady-state levels of p53, SOX2, and βIII-tubulin proteins were determined by Western blot analysis. Briefly, 50 μg of total protein extracts were separated on 12% (w/v) sodium dodecyl sulfate (SDS)- polyacrylamide gel electrophoresis (PAGE) and transferred onto nitrocellulose membranes using the Trans-blot Turbo Transfer System (BioRad). Uniform protein loading and transfer was confirmed by transient staining with 0.2% Ponceau S (Merck, Darmstadt, Germany). Membranes were blocked with 5% milk solution in Tris-buffered saline (TBS) for 1 h and incubated overnight at 4°C with primary mouse antibody reactive to p53 (Santa Cruz Biotechnology, sc-99, 1:200) or primary rabbit antibodies reactive to SOX2 (Merck, AB5603, 1:500) and βIII-tubulin (Biolegend, 801201, 1:500). After washing three times with TBS containing 0.2% Tween 20 (TBS-T), membranes were incubated with anti-rabbit or anti-mouse secondary antibodies conjugated with horseradish peroxidase (Bio-Rad; 1:5,000) for 2 h at room temperature. Finally, membranes were rinsed three times with TBS-T and the immunoreactive complexes detected by chemiluminescence using Immobilon Western Chemiluminescent HRP Substracte (Merck Millipore) or SuperSignal West Femto substrate (Thermo Fisher Scientific, Inc.) in a ChemiDoc MP System (Bio-Rad). Densitometric analysis of images was performed with the Image Lab software Version 6 Beta (Bio-Rad). Immunocytochemistry was performed to visualize intracellular levels of SOX2 and βIII-tubulin. NSCs were fixed with paraformaldehyde (4%, w/v) in PBS and then blocked for 1 h at room temperature in PBS containing 0.1% Triton™ X-100 (Roche Diagnostics, Mannheim, Germany), 1% FBS, and 10% normal donkey serum (Jackson ImmunoResearch Laboratories, Inc.). Subsequently, cells were incubated with primary mouse monoclonal antibodies reactive to βIII-tubulin (BioLegend®, MMS-435P) or SOX2 (R&D Systems®, MAB2018), at dilutions 1:500 and 1:100, respectively, in blocking solution, overnight at 4°C. After two washes, cells were incubated with anti-mouse secondary antibodies conjugated to Alexa Fluor® 568 (Molecular Probes®, Thermo Fisher Scientific, Inc.) for βIII-tubulin detection, or to DyLight® 488 (Molecular Probes®, Thermo Fisher Scientific, Inc.) for SOX2 detection, diluted 1:200 for 2 h at room temperature. Nuclei were stained with Hoechst 33258 (Sigma-Aldrich) at 50 μg/mL in PBS, for 5 min at room temperature. Samples were mounted using ProLong® Diamond Antifade Mountant (Molecular Probes®, Thermo Fisher Scientific, Inc.) and visualized with a Zeiss Axio Scope.A1 fluorescence microscope (Carl Zeiss Microscopy GmbH), equipped with an AxioCam HRm (Carl Zeiss Microscopy GmbH).

#### Cell Cycle Analysis

Cell cycle progression was evaluated using a standard staining procedure with propidium iodide (PI) (Fluka, Sigma-Aldrich) followed by flow cytometry. NSCs in differentiating conditions were treated with compound **1a** (12.5 μM) for the indicated time points. Next, cells were detached with accutase and collected by centrifugation at 800 *g* for 5 min, at 4°C. Cell pellets were resuspended in ice-cold PBS and fixed under gentle vortexing by dropwise addition of an equal volume of ice-cold 80% ethanol (−20°C), followed by 30 min on ice. Subsequently, samples were stored at 4°C for at least 18 h until data acquisition. For cell cycle analysis, cells were centrifuged at 850 g for 5 min, at 4°C, and pellets resuspended in RNase A (50 μg/mL, in PBS) and PI (25 μg/mL) and further incubated for 30 min, at 37°C. Acquisition of at least 10000 events per sample was performed using the Guava easyCyteTM Flow Cytometer (Merck Millipore). Data analysis was performed using Mod Fit LT 4.1 software (Verity Software House, Inc., Topsham, ME, USA).

#### Self-Renewal Assay

Self-renewal was measured through the cell-pair assay. Briefly, NSCs were plated in uncoated tissue culture plastic 12-well plates, at a density of 6,400 cells/cm^2^. After seeding, NSCs were grown in 1/2 EGF/bFGF containing medium supplemented or not (control) with low doses of **1a** (2.5 μM) for 24 h to avoid massive cell death under this cell density. Cells were then fixed in paraformaldehyde (4%, w/v) in PBS for 30 min at 4°C and then processed for immunocytochemistry against Sox2. In fact, Sox2, a transcription factor essential for maintaining self-renewal and pluripotency, tends to become cytoplasmic or disappear in dividing cells that start to differentiate (Graham et al., 2003; Thiel, 2013). In this regard, Sox2 pairs resulting from the division of a single stem/progenitor cell in undifferentiation conditions were categorized in the following groups: Sox2^+/+^; Sox2^+/−^; and Sox2^−/−^, which represent symmetrical division (self-renewal), asymmetrical division, and symmetrical division (differentiation), respectively. The number of progenitor pairs undergoing proliferative or differentiative cell divisions was determined by counting 60 pairs of cells, in control conditions or with **1a**, of at least three different experiments (Shen et al., 2002; Xapelli et al., 2013).

### Statistical Analysis

Student 2-tailed unpaired *t*-test was used to compare differences between two groups. *p* < 0.05 was considered statistically significant. Analysis and graphical presentation were performed with the GraphPad Prism Software version 5 (GraphPad Software, Inc., San Diego, CA, USA). Results are presented as mean ± standard error the mean (SEM) of at least three independent experiments.

## Results and Discussion

### Synthesis of Spiropyrazoline Oxindoles

**Figure 1:**
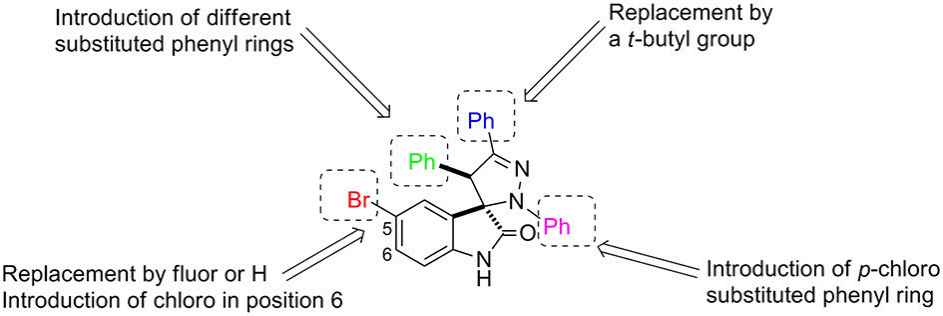
Structural modifications introduced in the spiropyrazoline oxindole scaffold.

To develop the chemical library (Figure 1) we focused our attention on structural modifications around the pyrazoline ring. In the oxindole ring we maintained fluor and chlorine atoms at positions 5 and 6, respectively, as the presence of these substituents in other spirooxindoles has led to potent MDM2 inhibitors (Wang et al., 2014; Bill et al., 2016; Ribeiro et al., 2017). In the pyrazoline ring we introduced different aromatic groups and the alkylic group *t*-butyl to mimic p53Leu23 and p53Phe19.

**Scheme 1:**
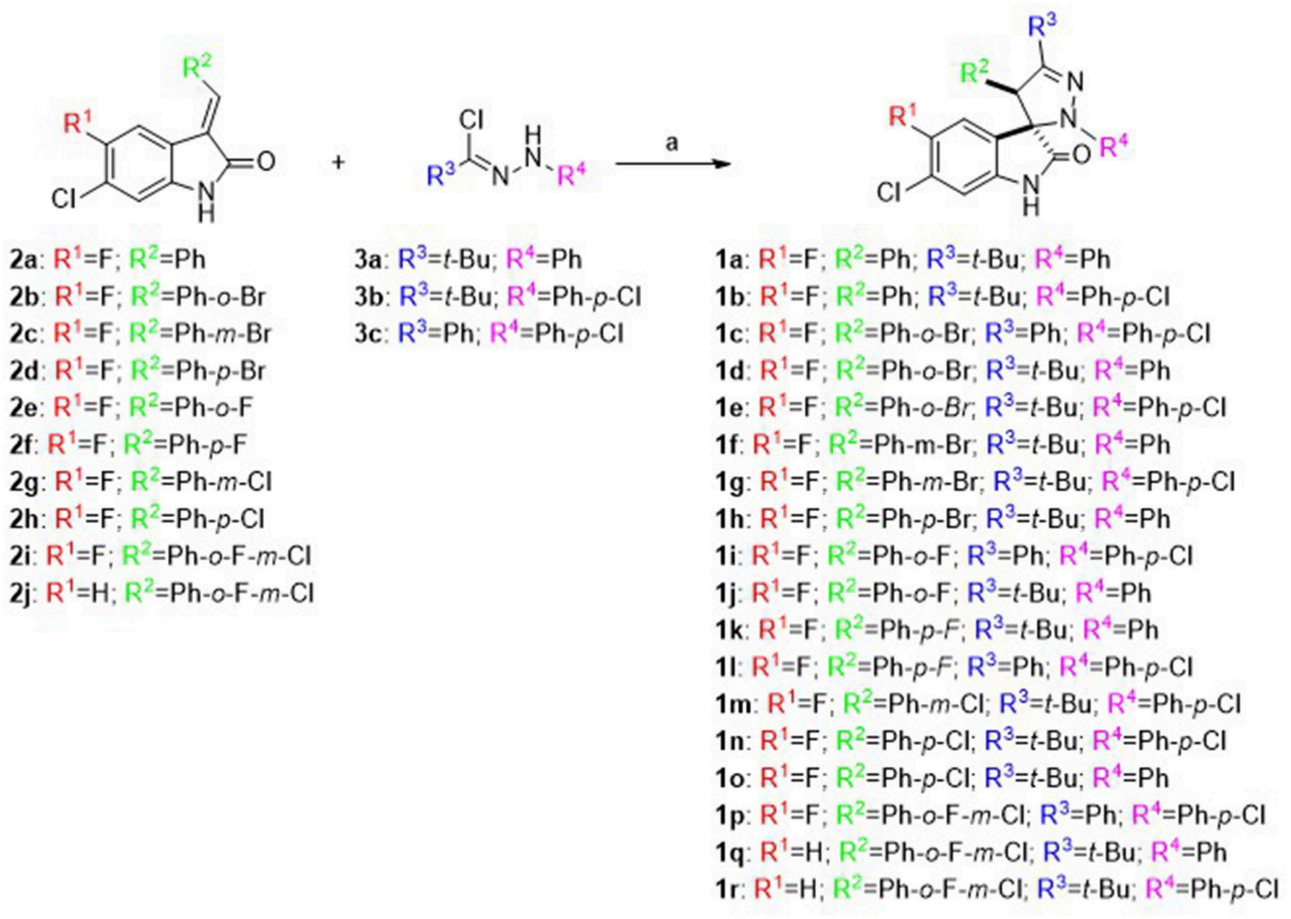
Synthesis of spiropyrazoline oxindoles **1a–r**: Reagents and conditions: (a) Et_3_N, CH_2_Cl_2_, rt, 16–24 h.

As shown in Scheme 1, spiropyrazoline oxindoles **1a**–**r** were synthesized by 1,3-dipolar cycloaddition reaction of 3-methylene indoline-2-ones **2a–j** and nitrile imines (formed *in situ* by dehydrohalogenation of hydrazonoyl chlorides **3a–c**), as previously reported (Monteiro et al., 2014). 3-Methylene indoline-2-ones **2a**–**j** were easily synthesized by aldolic condensation of indolin-2-ones with different commercially available benzaldehydes. The hydrazonoyl chlorides **3a**–**c** were synthesized by reacting the appropriate hydrazones with triphenylphosphine and carbon tetrachloride. The structure of all target compounds was confirmed by nuclear magnetic resonance (NMR) spectroscopy and mass spectrometry. The spiro and C-4′ carbon signals appear between 76.2–77.5 and 53.3–62.6 ppm, respectively. Moreover, the H-4′ signal appears between 4.39 and 5.67 ppm, in line with reported data (Wang et al., 2013; Nunes et al., 2017).

### Screening in Human Colorectal Carcinoma Cell Lines With and Without P53

To evaluate cytotoxicity and p53 selectivity of the synthesized compounds, spiropyrazoline oxindoles **1a**–**r** were screened using the isogenic pair of HCT116 human colorectal carcinoma cell lines differing only in the presence or absence of the *p53* gene. We were particularly interested in evaluating if these compounds could have a p53-driven effect without eliciting high levels of cell death.

We started our study by synthesizing and evaluating spiropyrazolines oxindoles containing fluor and chlorine atoms at positions 5 and 6, respectively, of the oxindole moiety, because previous studies had shown that these substituents increase the anti-proliferative activity against HCT116 colon cancer cell lines (Nunes et al., 2017). In substituent R^3^, we studied the effect of replacing an aromatic group by an alkyl group to better mimic the ^p53^Leu23. Moreover, we decided to study the anti-proliferative effect of introducing halogens in different positions of the aromatic rings (substituents R^2^ and R^4^). Finally, two spiropyrazolines oxindoles without a fluor substituent in the oxindole core were synthesized to determine the effect of the fluor atom on compound activity (Aguilar et al., 2017).

**Figure 2:**
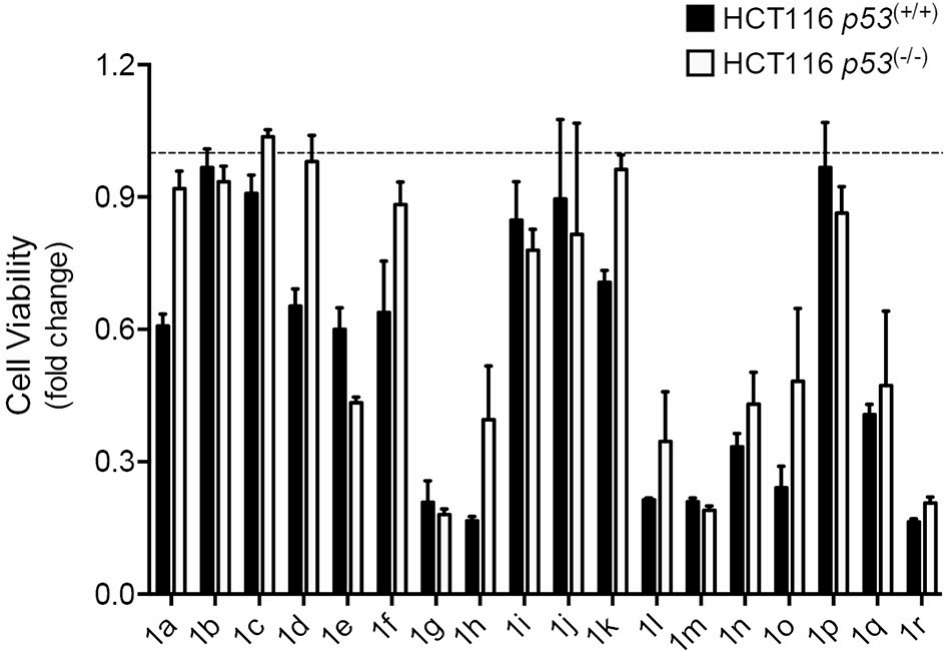
Spiropyrazolines oxindoles screen in HCT116 human colorectal carcinoma cells. HCT116 *p53*^(+/+)^ and HCT116 *p53*^(−/−)^ cells were exposed to spiropyrazoline oxindoles **1a–r** or vehicle (DMSO) at 12.5 μM for 72 h. Cell metabolism was evaluated by MTS assay. Results are expressed as mean ± SEM fold-change to vehicle treated cells from one experiment performed in duplicates. Dashed line represents vehicle control results.

As depicted in Figure 2, with exception of compounds **1b**, **1c**, and **1p** that did not alter cell viability, all the other compounds decreased cell viability in both cell lines when compared to DMSO controls. The results show that compounds **1g**, **1h**, **1l**, **1m**, and **1r** induced the highest decrease in cell viability, indicating high anti-proliferative activity, while compounds **1a**, **1d**, **1f**, **1h**, **1k, 1l**, and **1o** were the most selective compounds for the cancer cells expressing wild-type p53. In contrast with spiropyrazolines oxindoles **1f**, **1h**, **1k**, **1l**, and **1o** that only showed selectivity for HCT116 cells expressing p53 at 12.5 μM concentration, spiropyrazolines oxindoles **1a** and **1d** showed selectivity at both 12.5 and 25 μM (data not shown).

### Cell Viability and Differentiation in Mouse NSCs

**Figure 3:**
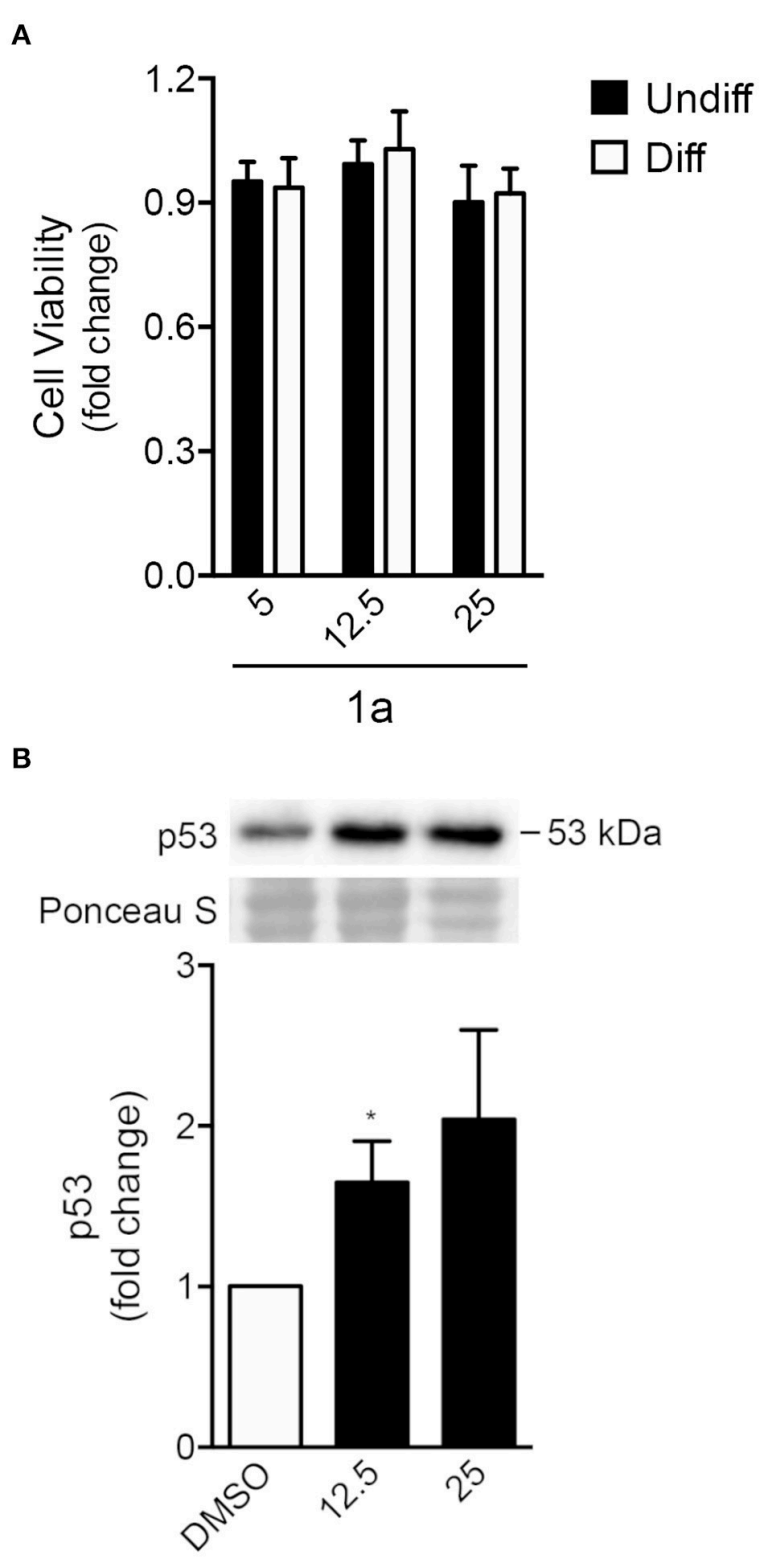
Spiropyrazoline oxindole **1a** does not induce significant toxicity but stabilizes p53 protein in NSCs. Mouse NSCs were treated with 5, 12.5, or 25 μM of compound **1a** or DMSO (control) for 24 h and collected for viability and Western blot assays as described in *Experimental*. **(A)** Cell viability as analyzed by MTS metabolism in self-renewing or differentiating NSCs after **1a** treatment **(B)** Representative immunoblots (top) and corresponding densitometry analysis (bottom) showing increased p53 protein levels in differentiating NSCs after **1a** treatment. Ponceau staining was used as loading control. Data represent mean ± SEM fold-change to control of at least three independent experiments. ^*^p < 0.05 from respective control by Student's *t*-test.

To explore the effect of spiropyrazoline oxindoles on neural differentiation potential, we selected the spiropyrazoline oxindole **1a**. This compound presented a moderate antiproliferative activity in the HCT116 *p53*^(+/+)^ colon cancer cell line (IC_50_ of 25 μM; data not shown). Further, compound **1a** was shown to be 1.5-fold more potent for HCT116 *p53*^(+/+)^ over HCT116 *p53* ^(−/−)^ cells. These two characteristics were indeed crucial for the choice of this compound since our main goal was to induce p53-mediated NSC differentiation rather than cell death. We began by determining the effect of **1a** in NSC viability by evaluating MTS metabolism in mouse NSCs at 24 h under self-renewing or differentiation conditions (Figure 3A). Adult NSCs are classified as self-renewing multipotent cells due to their capacity to differentiate in neurons, astrocytes and oligodendrocytes (Gage and Temple, 2013). In fact, it has been suggested that NSCs could be the cell of origin of primary brain tumors; however, through differentiation, they may also hold the key to brain tumor treatment (Germano et al., 2010; Zong et al., 2012). Here, we used an adherent model of NSCs which continuously expand by symmetrical division and are capable of tripotential differentiation (Pratt et al., 2000; Silva et al., 2006). In fact, this model is a more advantageous model relatively to neurospheres, with homogeneous composition and high neurogenic potential.

Our results showed that, under both self-renewing and differentiation conditions, NSC viability was not significantly affected by 24 h of **1a** treatment at either 12.5 or 25 μM concentrations (Figure 3A). We next investigated whether **1a** induced elevated levels of p53 even in the absence of significant NSC death. For that, p53 total levels were evaluated in **1a**-treated NSCs by Western blot. In fact, after 24 h of treatment, p53 steady-state levels significantly increased in NSCs, when compared with untreated NSCs (Figure 3B), confirming that **1a** induced p53 stabilization. The increase of p53 stability by **1a** was corroborated by exposing NSCs to compound **1a** or vehicle with 100 μg/ml cycloheximide for a maximum of 60 min. Indeed, in this experiment we also observed increased levels of p53 in cells treated with **1a** when compared with control cells treated with DMSO (Supplementary Datasheet 2, Figure R1). Although NSC death was not observed, it remained unclear whether **1a**-induced p53 was associated with neural differentiation.

**Figure 4:**
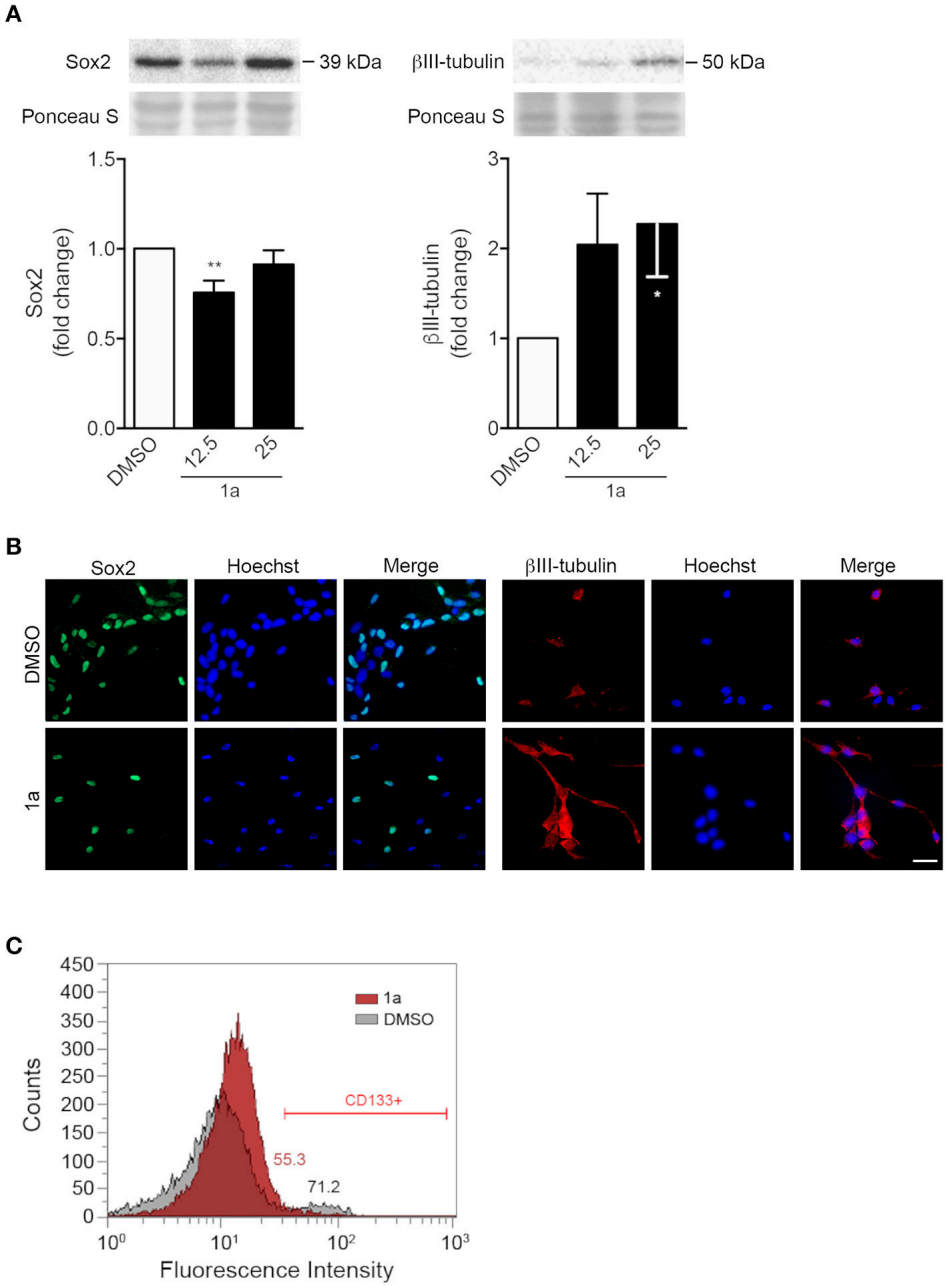
Spiropyrazoline oxindole **1a** decreases stemness and increases differentiation of NSCs. Mouse NSCs were grown in differentiation medium, incubated with 12.5 or 25 μM **1a** or DMSO (control) for 24 h and collected for Western blot and immunocytochemistry as described in *Experimental*. **(A)** Representative immunoblots (top) and corresponding densitometry analysis (bottom) showing decreased Sox2 (left) and increased βIII-tubulin (right) protein levels after **1a** treatment. Ponceau staining was used as loading control. Data represent mean ± SEM of at least three independent experiments. ^*^p < 0.05 and ^**^p < 0.005 from respective control by Student's *t*-test. **(B)** Representative images of immunofluorescence detection of Sox2 (left) and βIII-tubulin (right) after 24 h of **1a** treatment at 12.5 μM. Nuclei were stained with Hoechst 33258. Scale bar, 25 μm. **(C)** Flow cytometry histograms showing surface marker expression of stemness-associated CD133, after treatment with vehicle (gray) or **1a** at 12.5 μM (red) for 24 h, and respective quantification of CD133-positive cells mean fluorescence intensity.

To better understand the impact of compound **1a** in modulating differentiation of NSCs, we evaluated the steady-state levels of proteins Sox2 and βIII-tubulin upon exposure to **1a** (Figure 4). Sox2 is a transcription factor that plays a key role in the self-renewal process of stem cells, being considered a hall-mark of pluripotency, or multipotency in the case of NSCs. In contrast, βIII-tubulin is a well-known marker of neural differentiation, found almost exclusively in neuron microtubules. Therefore, while Sox2 levels are expected to gradually decrease throughout NSC differentiation, βIII-tubulin is expected to increase.

Our results showed that 24 h of **1a** incubation induced a significant decrease in Sox2. Further, a significant increase of 60% was detected for the neuronal specific marker βIII-tubulin in differentiating NSCs, as assessed by Western blot (Figure 4A). Importantly, immunocytochemistry was in agreement with immunoblotting results (Figure 4B). Since p53 has been shown to repress the transcription of the cell surface protein CD133 (Park et al., 2015), the best-characterized stemness marker in distinct solid tumors, we also assessed the effect of **1a** on this specific p53 target molecule. Notably, our results revealed that **1a** treatment diminished the expression of CD133 marker in NSCs (Figure 4C). Since this stemness marker has been correlated with CSC tumor-initiating capacity (Curley et al., 2009), this data also suggests that this novel spiropyrazoline oxindole may impact on self-renewal potential.

**Figure 5:**
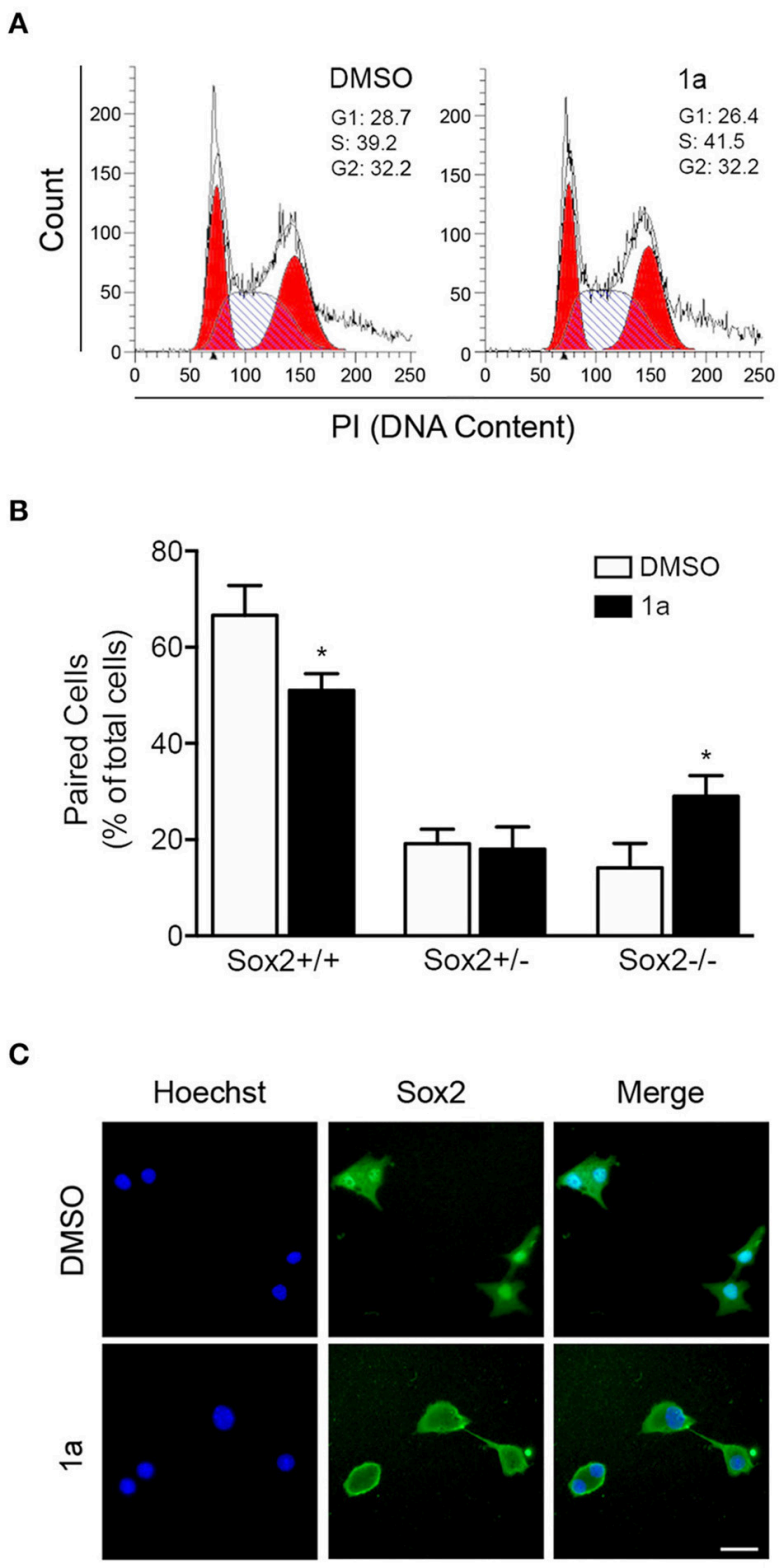
Spiropyrazoline oxindole **1a** decreases NSC self-renewal through a cell cycle independent manner. Mouse NSCs were treated with compound **1a** at 12.5 μM or DMSO (control) for 24 h and collected for flow cytometry and cell-pair assays as described in *Experimental*. **(A)** Effect of compound **1a** on cell cycle progression. Cellular DNA was stained with propidium iodide (PI) in differentiation conditions to determine cell cycle distribution. **(B)** Representative quantification data of paired cells Sox2-positive and -negative, in low EGF/bFGF containing medium after **1a** treatment. **(C)** Representative microscopy images of undifferentiated NSC pairs labeled with Hoechst 33258 for nuclei staining and anti-Sox2 antibody after **1a** treatment. Scale bar, 25 μm. Results are expressed as mean ± SEM fold-change for at least three different experiments. ^*^p < 0.05 from control by Student's *t*-test.

To go deeper into the mechanisms by which this p53-stabilizing molecule impacts on NSCs differentiation status, we evaluated cell cycle progression of NSCs after compound **1a** treatment. Curiously, flow cytometry analysis of NSC DNA content indicated that this spiropyrazoline oxindole did not affect cell cycle progression. In fact, 24 h of compound **1a** did not induce significant alterations in G1, S, and G2 phases in either self-renewing and differentiating NSCs (Figure 5A). Similar results were obtained after 4, 8, and 48 h of **1a** treatment (data not shown).

To further understand the mechanism of **1a**-induced neural differentiation, and taking into consideration of the **1a**-induced effects on CD133 expression, we used the *in vitro* self-renewal cell-pair assay to assess how multipotent and non-multipotent cells divide in the presence or absence of **1a**. The cell-pair assay was performed by culturing NSCs in low density and low percentage of growth factors, and evaluating the expression of the multipotent marker Sox2 in mitotic cell pairs by immunocytochemistry. Mitotic cell pairs, resulting from the division of a single stem/progenitor cell, were then counted and categorized in three groups according to their Sox2 nuclear localization: in both daughter cells (Sox2^+/+^), in only one daughter cell (Sox2^+/−^), in none of them (Sox2^−/−^). In fact, it has been described that subcellular localization of Sox2 can be either cytoplasmic or nuclear, depending on the differentiation levels (Avilion et al., 2003; Baltus et al., 2009); when cells differentiate, SOX2 becomes cytoplasmic or is lost altogether. Interestingly, lower levels of nuclear Sox2^+^/Sox2^+^ symmetrical divisions were detected after 24 h of **1a** incubation, when compared with control cells (Figures 5B,C). Accordingly, Sox2^+^/Sox2^−^ asymmetric and Sox2^−^/Sox2^−^ symmetric divisions happened more frequently in **1a**-treated NSCs. In addition, to corroborate the involvement of p53 in **1a**-repressed symmetric divisions, we assessed cell-pair assay in HCT116 p53^(+/+)^ and HCT116 *p53*^(−/−)^ cell lines after **1a** treatment. Our results showed that 24 h of **1a** significantly reduced symmetric stem cell divisions in HCT116 *p53*^(+/+)^, but the effect of 1a was completely abolished in HCT116 *p53*^(−/−)^ (Supplementary Datasheet 2, Figure R2). Taken together, this data indicates that this p53 stabilizer molecule regulates the neural differentiation process by targeting p53 and interfering with the balance between symmetric and asymmetric divisions of NSCs through a cell cycle-independent mechanism. Of note, the asymmetric cell division cycle has been shown to be orchestrated by a complex gene-protein regulatory network, often independent of the cell-cycle progression mode (Okamoto et al., 2016).

### Differentiation and Chemotherapy Sensitization of Glioma Cells

**Figure 6:**
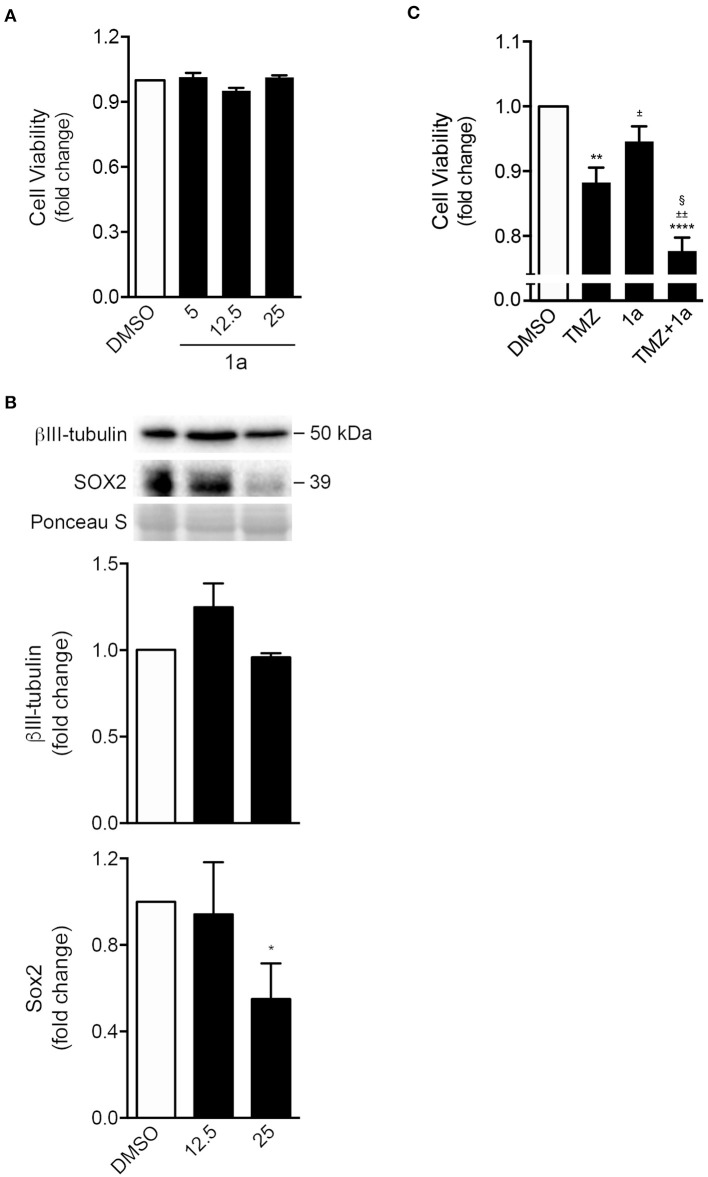
Spiropyrazoline oxindole **1a** reduces stemness of GL-261 mouse glioma cells while sensitizes conventional chemotherapeutic-induced cell death. GL-261 cells were treated with compound **1a** or DMSO (control) for 24 h and collected for MTS and Western blot analysis. **(A)** Cell viability as analyzed by MTS metabolism in GL-261 cells treated with 5, 12.5, or 25 μM of compound **1a**. **(B)** Representative immunoblots (top) and corresponding densitometry analysis (bottom) of Sox2 and β-III tubulin protein levels after 24 h of 12.5 or 25 μM of **1a** treatment. Ponceau staining was used as loading control. **(C)** Cell viability as analyzed by MTS metabolism in GL-261 cells co-treated with **1a** (12.5 μM) and the chemotherapeutic agent TMZ (400 μM) for 24 h. Data represent mean ± SEM of at least three independent experiments. ^*^p < 0.05, ^**^p < 0.005, and ^****^p < 0.0001 from DMSO; ^±^*p* < 0.01 and ^§^*p* < 0.0001 from TMZ-treated cells; ^±±^*p* < 0.0001 from **1a**-treated cells by one-way ANOVA followed by Tukey's multiple comparison test. TMZ, temozolomide.

To clarify the potential of spiropyrazoline oxindole **1a** in regulating tumor neural cell fate, we evaluated the effect of **1a** incubation in the GL-261 mouse glioblastoma cell line. As depicted in Figure 6A, exposure of GL-261 cells to **1a** for 24 h marginally changed cell survival, indicating no significant toxic effects. Therefore, based on the available literature regarding the relevance of differentiation status in tumor chemotherapy sensitization, and the unique neurogenic properties of **1a** here described, we hypothesized that **1a** could also interfere with glioma cell differentiation. Indeed, Western blot analysis showed that treatment with 25 μM of **1a** for 24 h significantly decreased Sox2 protein levels by ~40% (Figure 6B), suggesting that this stemness-related protein may also be a potential target of **1a** in neural tumor cells. The levels of βIII-tubulin, in turn, were only slightly increased with a lower dose of **1a**.

Finally, and because drug combination is widely used to achieve treatment efficacy in several types of tumors (Patties et al., 2016), we evaluated the potential of compound **1a** in sensitizing tumor cells to the conventional chemotherapeutic temozolomide (TMZ). The main goal is to achieve a synergistic therapeutic effect accompanied by a decrease of toxicity, and diminished drug resistance. Of note, TMZ is the most used chemotherapeutic agent in the treatment of primary tumors of the central nervous system (Friedman et al., 2000). As shown in Figure 6C, when GL-261 cells were incubated with a combination of **1a** and TMZ, for 24 h, cell viability significantly declined when compared to TMZ treatment alone.

## Conclusions

The antiproliferative activity of eighteen spiropyrazoline oxindoles was assessed in the HCT116 human colorectal carcinoma isogenic pair, with and without p53. From this initial screening, we selected one compound to study the effect of the spiropyrazoline oxindole scaffold on the induction of other p53-mediated outcomes in NSC differentiation. Under both self-renewing and differentiation conditions, NSC viability was not significantly affected by this compound. The mechanism of action of spiropyrazoline oxindoles as inducers of neural differentiation by a p53-dependent pathway was studied in more detail. Our results showed that p53 steady-state levels in NSCs significantly increased after 24 h of **1a** treatment, when compared with untreated conditions. Additionally, this spiropyrazoline oxindole induced a significant decrease in Sox2 levels and a significant increase of the neuronal βIII-tubulin marker in differentiating NSCs, while also reducing symmetric renewal divisions in these cells. Furthermore, to clarify the potential of spiropyrazoline oxindoles in regulating tumor neural cell fate, the effect of spiropyrazoline oxindole **1a** in cell viability of the tumoral cell line GL-261 was evaluated. MTS metabolism revealed that there were almost no changes in human glioma cell survival at 24 h after **1a** incubation, indicating no toxic effect of this small molecule even in neural tumor cells. In contrast, **1a** treatment negatively regulated the GL-261 stemness potential. Combined treatment with TMZ led to a decline of cell viability of the neural tumor cell line when compared with treatment with TMZ alone. These results strongly support the idea that **1a** offers great potential as a drug development tool in combined therapies for brain cancer, possibly with less side effects. In conclusion, we successfully identified novel spiropyrazoline oxindoles that act as p53 stabilizers, with one redirecting cells toward neural differentiation rather than cell death. To our knowledge, this is a unique derivative with the ability to selectively influence p53 and sensitize neural cells for chemotherapy through stimulation of differentiation.

## Author Contributions

DS performed the synthesis and characterization of the target compounds. JA and DS performed the biological assays and analyzed the data. CR reviewed the manuscript. SS and MS conceived and designed the study, analyzed the data, and critically reviewed the manuscript. The manuscript was written and approved by all authors for publication.

### Conflict of Interest Statement

The authors declare that the research was conducted in the absence of any commercial or financial relationships that could be construed as a potential conflict of interest.
