# Corrigendum: A Novel Small Molecule p53 Stabilizer for Brain Cell Differentiation

**DOI:** 10.3389/fchem.2019.00282

**Published:** 2019-04-24

**Authors:** Joana D. Amaral, Dário Silva, Cecília M. P. Rodrigues, Susana Solá, Maria M. M. Santos

**Affiliations:** Research Institute for Medicines (iMed.ULisboa), Faculty of Pharmacy, Universidade de Lisboa, Lisbon, Portugal

**Keywords:** antiproliferative agents, brain tumor, cell differentiation, p53, spirooxindole

In the original article, there was a mistake in Scheme 1 as published. R1 in compounds 1j, 1k, 1l, 1m, 1n, 1o, and 1p should be “F” instead of “H”. The corrected Scheme 1 appears below.

**Scheme 1:**
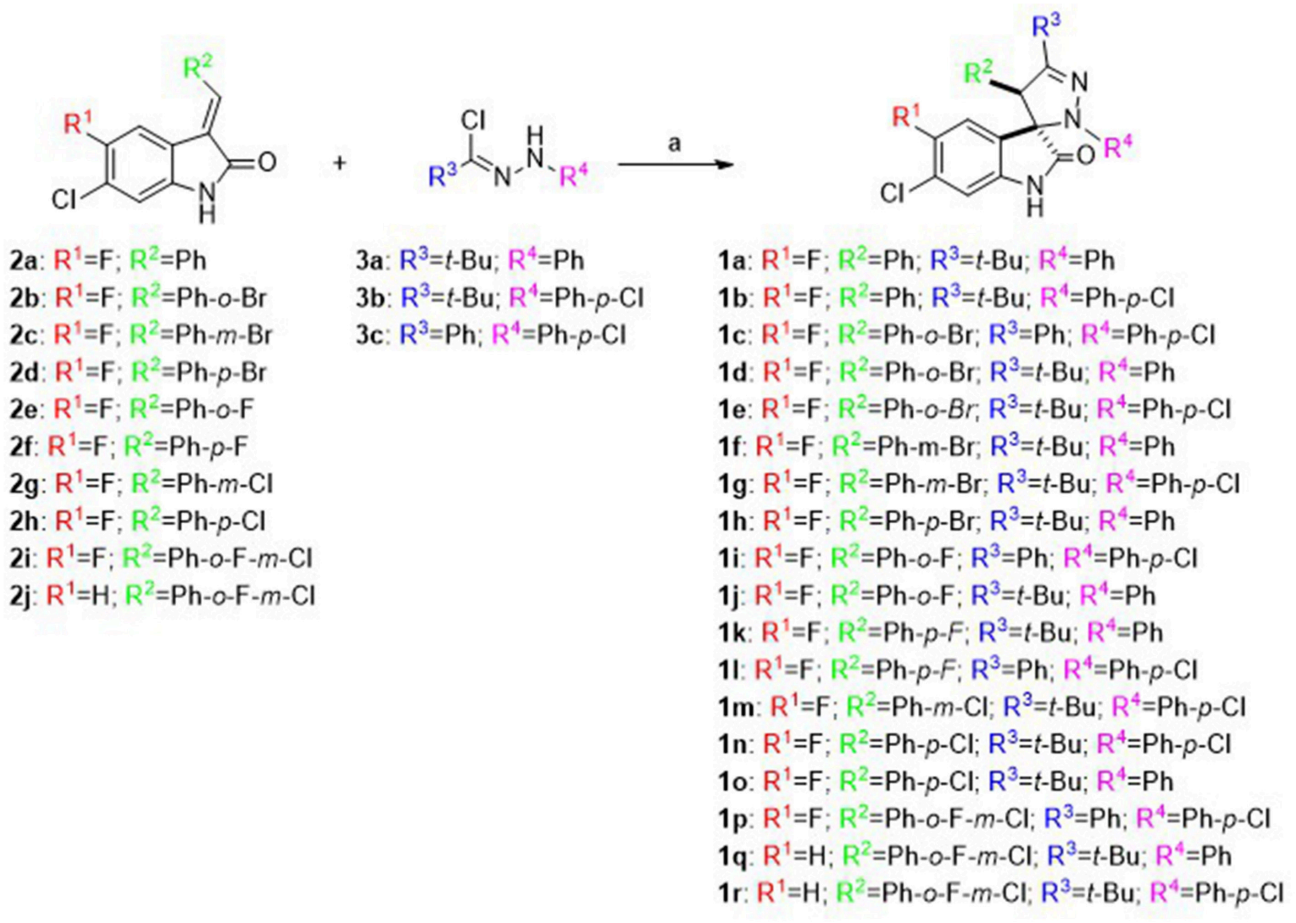
Synthesis of spiropyrazoline oxindoles **1a–r**: Reagents and conditions: (a) Et_3_N, CH_2_Cl_2_, rt, 16–24 h.

The authors apologize for this error and state that this does not change the scientific conclusions of the article in any way. The original article has been updated.

