# Supplementary material for: A Novel Small Molecule p53 Stabilizer for Brain Cell Differentiation
Source: Front Chem. 2019 Jan 31;7:15. doi: 10.3389/fchem.2019.00015 (PMC6365904; doi:10.3389/fchem.2019.00015)
Supplement: Supplementary file 1 [file Data_Sheet_1.docx]

Supplementary Material

A novel small molecule p53 stabilizer for brain cell differentiation

Joana D. Amaral, Dário Silva, Cecília M. P. Rodrigues, Susana Solá*, Maria M. M. Santos*

*** Correspondence:** Dr. Susana Solá (susana.sola@ff.ulisboa.pt); Dr. Maria M. M. Santos (mariasantos@ff.ulisboa.pt)

Figure 1. ^1^H NMR of compound **1a**.

Figure 2. ^13^C NMR of compound **1a**.
