# Supplementary material for: A Novel Small Molecule p53 Stabilizer for Brain Cell Differentiation
Source: Front Chem. 2019 Jan 31;7:15. doi: 10.3389/fchem.2019.00015 (PMC6365904; doi:10.3389/fchem.2019.00015)
Supplement: Supplementary file 2 [file Data_Sheet_2.docx]

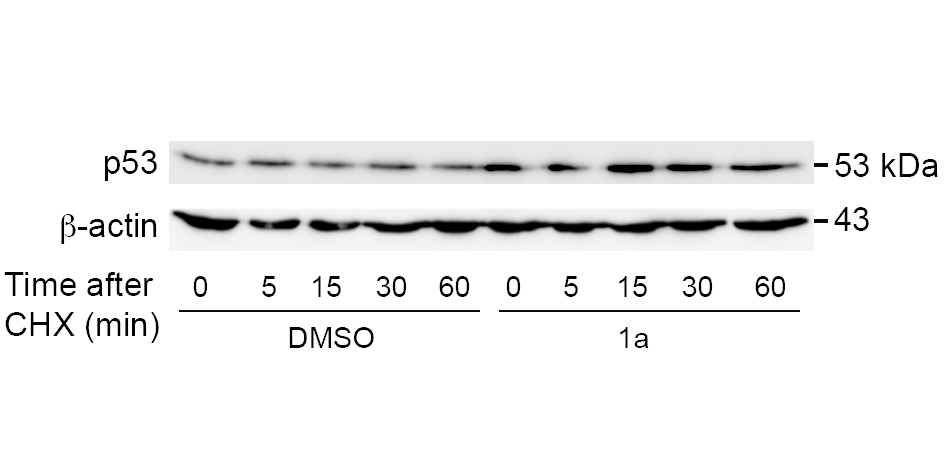


**Figure R1**. Spiropyrazoline oxindole **1a** increases p53 stability. Mouse neural stem cells (MNSCs) were plated in 60-mm dishes at 4.2x10^4^ cells/cm^2^. Twenty-four hours after plating, cells were treated with vehicle or 12.5 μM of **1a** in differentiation medium for additional 24h. Cells were chased with cycloheximide (Sigma-Aldrich) at a final concentration of 100 μM/mL. At the indicated time points after the treatment with cycloheximide, cells were harvested, and whole-cell lysates were processed for immunoblot analysis with the anti-p53 antibody (sc-99). β-actin was used as protein loading control.


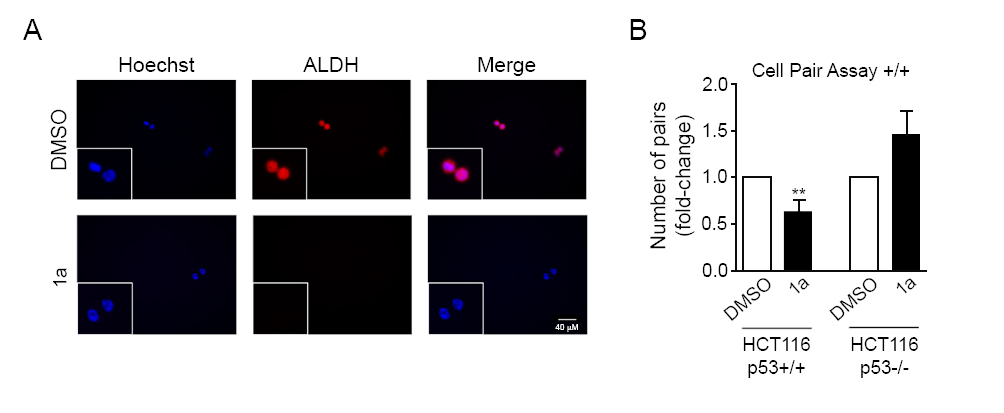


**Figure R2**. Spiropyrazoline oxindole **1a** disrupts self-renewal in HCT116 p53+/+ cells. HCT116 p53+/+ and p53-/- cells were incubated with 2.5 μM **1a** for 24 h. DMSO was used as vehicle control. **(A)** Representative microscopy images of HCT116 p53+/+ cell pairs labeled with Hoechst 33258 and anti-ALDH1/2 antibody (sc-50385) after **1a** treatment. Scale bar, 40 µm. **(B)** Representative quantification data of paired cells ALDH-positive, in low EGF/bFGF containing medium after **1a** treatment. Results are expressed as mean ± SEM fold-change to vehicle control of at least 3 different experiments. **p < 0.01 from DMSO.
